# MicroRNA Expression in Formalin-fixed Paraffin-embedded Cancer Tissue: Identifying Reference MicroRNAs and Variability

**DOI:** 10.1186/s12885-015-2030-2

**Published:** 2015-12-29

**Authors:** Mogens Karsbøl Boisen, Christian Dehlendorff, Dorte Linnemann, Nicolai Aagaard Schultz, Benny Vittrup Jensen, Estrid Vilma Solyom Høgdall, Julia Sidenius Johansen

**Affiliations:** Department of Oncology, Herlev and Gentofte Hospital, Herlev Ringvej 75, DK-2730 Herlev, Denmark; Statistics, Bioinformatics and Registry, Danish Cancer Society Research Center, Copenhagen, Denmark; Department of Pathology, Herlev and Gentofte Hospital, Herlev, Denmark; Department of Surgical Gastroenterology, Rigshospitalet, Copenhagen, Denmark; Department of Medicine, Herlev and Gentofte Hospital, Herlev, Denmark; Faculty of Health and Medical Sciences, University of Copenhagen, Copenhagen, Denmark

**Keywords:** microRNA, FFPE, Colorectal cancer, Pancreatic cancer, Biomarkers, Normalization

## Abstract

**Background:**

Archival formalin-fixed paraffin-embedded (FFPE) cancer tissue samples are a readily available resource for microRNA (miRNA) biomarker identification. No established standard for reference miRNAs in FFPE tissue exists. We sought to identify stable reference miRNAs for normalization of miRNA expression in FFPE tissue samples from patients with colorectal (CRC) and pancreatic (PC) cancer and to quantify the variability associated with sample age and fixation.

**Methods:**

High-throughput miRNA profiling results from 203 CRC and 256 PC FFPE samples as well as from 37 paired frozen/FFPE samples from nine other CRC tumors (methodological samples) were used. Candidate reference miRNAs were identified by their correlation with global mean expression. The stability of reference genes was analyzed according to published methods. The association between sample age and global mean miRNA expression was tested using linear regression. Variability was described using correlation coefficients and linear mixed effects models. Normalization effects were determined by changes in standard deviation and by hierarchical clustering.

**Results:**

We created lists of 20 miRNAs with the best correlation to global mean expression in each cancer type. Nine of these miRNAs were present in both lists, and miR-103a-3p was the most stable reference miRNA for both CRC and PC FFPE tissue. The optimal number of reference miRNAs was 4 in CRC and 10 in PC. Sample age had a significant effect on global miRNA expression in PC (50 % reduction over 20 years) but not in CRC. Formalin fixation for 2–6 days decreased miRNA expression 30–65 %. Normalization using global mean expression reduced variability for technical and biological replicates while normalization using the expression of the identified reference miRNAs reduced variability only for biological replicates. Normalization only had a minor impact on clustering results.

**Conclusions:**

We identified suitable reference miRNAs for future miRNA expression experiments using CRC- and PC FFPE tissue samples. Formalin fixation decreased miRNA expression considerably, while the effect of increasing sample age was estimated to be negligible in a clinical setting.

**Electronic supplementary material:**

The online version of this article (doi:10.1186/s12885-015-2030-2) contains supplementary material, which is available to authorized users.

## Background

MicroRNAs (miRNAs) are ~22 nucleotides long non-protein-coding RNAs involved in post-transcriptional regulation of gene expression [1, 2]. Mature miRNAs join the RNA-induced silencing complex (RISC) in the cytoplasm and bind to messenger RNAs (mRNAs), whereby they block translation or induce degradation of the mRNA transcript. Each miRNA targets specific genes through sequence complementarity between the miRNAs "seed" region (nucleotides 2–7) and a miRNA recognition element (MRE) in the mRNA, most often located in the 3'-untranslated region (3'UTR). More than 2,500 mature human miRNA sequences have been annotated so far (http://www.mirbase.org) [3]. Because of the targeting of the miRNA seed region to a specific 7-nucleotide MRE in the mRNA, each miRNA can potentially regulate the expression of hundreds of genes. Indeed, it is estimated that most of the protein-coding genes are regulated by miRNAs [2, 4]. Accordingly, most, if not all, developmental, physiological and disease processes, such as cancer, are regulated by miRNAs [5, 6]. MiRNAs are involved in all the hallmark capabilities of cancer [7–9]. Deregulation of miRNA expression is associated with cancer development, and changes in miRNA expression are associated with survival in patients with cancer [9].

MiRNAs have been investigated intensely as potential biomarkers in cancer. A commonly used method for determination of miRNA expression is the reverse transcription quantitative polymerase chain reaction (RT-qPCR). RT-qPCR can be utilized to measure either single- or multiple miRNAs per experiment. One of the most important and challenging issues in miRNA expression experiments is normalization. The purpose of normalization is to remove as much non-biological variation, "noise" and bias, from the data as possible and to make it possible to compare results within or between experiments. In large microarray studies in which the expression of hundreds of miRNAs is measured, global mean normalization is the gold standard [10]. This normalization method uses the average expression of all miRNAs in each sample for normalization. In experiments with a smaller number of miRNAs, global mean normalization is not an option, and instead reference genes are needed for normalization. Traditionally, small nuclear- or nucleolar RNAs like *RNU6B* have been used for normalization in miRNA experiments. Yet, these have been shown to be inferior to the use of stably expressed global mean-associated miRNAs as reference genes [11–13]. Only a few studies have identified suitable reference miRNAs for use in frozen blood or tissue samples [10–15], and no published studies have identified reference miRNAs in FFPE tissue in an unbiased manner. Because miRNAs are highly tissue specific [14], reference miRNAs need to be validated within each tissue and tumor type, and for some prevalent malignancies like pancreatic cancer (PC), no publications regarding suitable reference miRNAs exist.

In the present study, we sought to identify stable reference miRNAs useful for normalization of RT-qPCR-determined miRNA expression in FFPE tissue samples from patients with colorectal cancer (CRC) and PC, and also to quantify the sources of variability associated with measurements of miRNA expression in archival FFPE samples.

## Methods

### Cohorts and clinical data

The miRNA measurements used for identifying reference miRNAs in this paper were produced in two previously published studies of CRC and PC [16–18]. For details regarding the clinical study populations, readers are referred to these papers. Importantly, all of the samples used were resected before any systemic treatment was initiated. Briefly, the 203 CRC samples (“CRC cohort”) were collected retrospectively from patients with metastatic CRC (mCRC) who had started first line treatment with capecitabine, oxaliplatin, and bevacizumab from 2006 to 2011 at one of 10 departments of oncology in Denmark. The endpoint overall survival (OS) was measured from initiation of first-line treatment to death from any cause. The vital status of all patients was updated in July 2013. The 256 PC samples (“PC cohort”) were retrospectively collected from patients undergoing surgery for pancreatic ductal adenocarcinoma or ampullary adenocarcinoma at the Department of Surgical Gastroenterology, Herlev University Hospital, from 1976 to 2008. Control tissue samples from resected normal pancreas (*n* = 20) and chronic pancreatitis (*n* = 20) were also included. OS in this cohort was measured from surgical resection to death from any cause, and participant vital status was updated October 2010. Patients in both cohorts who were alive at the time of last vital status update were censored. The 37 methodological samples were all from patients with CRC who had undergone surgery at the Department of Surgical Gastroenterology at Herlev University Hospital. The methodological samples that were used for the comparison of frozen- and FFPE tissue were anonymized samples acquired from the Danish CancerBiobank at Herlev University Hospital.

### Tissue sample handling and -preparation

All the samples in the CRC- and PC cohorts were FFPE samples from primary tumors handled according to the standard procedures at each local department of pathology. In general, resected tumor specimens were transported to the pathology department right after surgery. The specimens were then inspected and described by the pathologist, and the tumors were fixed in 10 % formalin-fixation solution for at least 48 hours, most often 2–3 days, but sometimes up to 5 days. After fixation, tumors sections were embedded in paraffin, and then stored at room temperature in a dry environment.

A collection of methodological samples from nine different CRC tumors was also used. These samples were treated differently: frozen or formalin fixed and paraffin embedded and serially sectioned. Within 30 minutes after surgery, tumor tissue was partitioned into three or four sections. One tumor section was immediately frozen in liquid isopentane. When frozen, the tumor was transferred to a container and kept in the freezer at -80 °C. The remaining tumor sections were fixed in formalin for 2, 3, or 6 days and then embedded in paraffin and kept at room temperature.

The diagnosis of carcinoma was confirmed by an experienced gastro-intestinal pathologist (DL) by review of a 3-μm hematoxylin and eosin (HE)-stained section from each tumor block. Three 10-μm sections were then cut from each tumor block without micro- or macro-dissection, and the sections were placed in sterile Eppendorf tubes. An overview of how the individual methodological samples were handled is provided in Table S1 in Additional file 1. From tumors 1–3, adjacent FFPE sections were cut and placed in two separate tubes. From tumors 4–9, sections were cut from both frozen samples and from FFPE tissue samples that were fixed for 2 to 6 days. From tumor 4, additional adjacent FFPE sections were cut and placed in five separate tubes.

The tissue sectioning was performed by experienced laboratory technicians at the Department of Pathology, Herlev University Hospital.

### MiRNA purification

All miRNA purification and expression analyses procedures were performed by the biotech service provider AROS Applied Biotechnology, Aarhus, Denmark (www.arosab.com) using commercially available reagents. The company was blinded to all clinical information.

For the CRC cohort and the methodological samples, RNA was purified using the miRNeasy FFPE Kit (Qiagen, Hilden, Germany) according to the manufacturer’s instructions (miRNeasy FFPE Handbook September 2010, www.qiagen.com). Briefly, samples were deparaffinized and then lysed with proteinase K digestion followed by heat treatment. After centrifugation, the supernatant was recovered and treated with DNase. After mixing with buffer and ethanol, part of the mixture was transferred to an RNeasy MinElute spin column where total RNA was bound. After washing, the RNA was eluted and normalized to 70 ng/μl manually. For the PC cohort samples, RNA was purified using the High Pure miRNA Isolation Kit (Roche, Basel, Switzerland) according to the manufacturer’s instructions. Briefly, the tissue sections were deparaffinized in xylene and ethanol, then treated with proteinase K, and finally RNA was isolated using the one-column spin column protocol for total RNA. After washing, the RNA was eluted and an aliquot was normalized to 133 ng/μl. A few samples (approximately 10) were below 133 ng/μl and were therefore concentrated by speed vac. The purity and concentration of RNA were assessed by absorbance spectrophotometry on a NanoDrop 8000 (Thermo Fisher Scientific, Waltham, MA, USA). Samples with a 260/280 nm absorbance ratio below 1.8 were discarded and new sections from the corresponding tissue block were cut and purified, if possible. Purified samples were stored at -80 °C until they were used for miRNA expression analysis.

### MiRNA reverse transcription and expression analysis

The TaqMan® Array Human MicroRNA A + B Cards Set version 3.0 (Applied Biosystems/Life Technologies, Carlsbad, CA, USA) was used to quantify expression of 754 miRNAs in the CRC cohort samples and in the methodological samples. The same array in version 2.0 was used for the PC cohort samples. The A-card (377 miRNAs) contained the same miRNA assays in the two versions, whereas there were minor differences between the B-cards. The instructions and reagents from the manufacturer were used in all steps (http://www.lifetechnologies.com/). Briefly, the procedure utilized for the array analysis was as follows. RNA was reverse transcribed (RT) using the TaqMan® MicroRNA Reverse Transcription Kit into cDNA in two multiplex reactions each containing 3 μl of the small RNA preparation, corresponding to 200 ng total RNA, and either Megaplex RT Primer Pool A or Pool B in a total reaction volume of 7.5 μl. The RT reaction was run at 16 °C for 2 min, 42 °C for 1 min, and 50 °C for 1 sec for 40 cycles, then at 85 °C for 5 min, and held at 4 °C. Prior to loading the arrays, a 12-cycle pre-amplification reaction was performed using 2.5 μl cDNA in a 25-μl reaction and using Megaplex PreAmp Primers Pool A or B. The preamplification was run at 95 °C for 10 min, 55 °C for 2 min, 72 °C for 2 min, and then 12 cycles at 95 °C for 15 sec and 60 °C for 4 min, and finally 99.9 °C for 10 min and held at 4 °C. The preamplified solution was then diluted with 75 μl 0.1x TE buffer to a total volume of 100 μl. Each of the arrays was loaded with 1/50 (8 μl) of the preamplification reaction which was mixed with TaqMan Gene Expression Master Mix in a total reaction volume of 800 μl and run on the 7900HT Fast Real-Time PCR System. The PCR reaction was run using the same program as for the RT. The quantification cycle (Cq) was defined as the fractional cycle number at which the fluorescence passed the fixed threshold. All raw C_q_ values >32 were discarded. MiRNA expression values were transformed to 40-C_q_ so that higher values corresponded to higher expression. Data from samples that had been analyzed in spite of a 260/280 nm absorbance ratio <1.8 were removed from the final data set. Data from methodological samples with a 260/230 nm absorbance ratio <1.5 were also removed.

The technical replicates were all from the same purification, and they were analyzed on the same day on the same machine, consecutively.

### Statistical analysis

Normalization: Global mean miRNA expression was defined as the sample-wise arithmetic mean C_q_ of all included miRNA measurements. In analyses in which low expression measurements and inconsistently expressed miRNAs were removed, only the remaining measurements were used to calculate global mean. Global mean normalization was performed as previously described [10]. Briefly, the mean of all C_q_ values for sample “i” (global mean) was subtracted from each individual C_q_ value from sample “i”. Normalization using reference miRNAs was performed in a similar manner using the arithmetic mean of the reference miRNAs instead of the global mean.

#### Identification of reference miRNAs (CRC cohort and PC cohort)

Calculations were performed separately for each tumor type. Only miRNAs with less than 5 % missing values were considered. Missing/undetermined = no signal after 40 PCR cycles. A list of 20 miRNAs with the best correlation to global mean miRNA expression was identified using Spearman's rank correlation coefficient, *r*_*s*_. The association between the expression of each of the 20 miRNAs and OS was investigated using a Cox proportional hazards (CPH) model [19, 20] to test if the expression of individual candidate reference miRNAs was independent of prognosis. The stability measure *M* was calculated for each of the 20 miRNA according to the formula by Vandesompele et al*.*[21]. If more than one miRNA from the same miRNA family was included in the list, only the miRNA with the lowest *M* value was kept (miRNA family list found at http://www.mirbase.org/ftp.shtml). Removing miRNAs from the same family is recommended because miRNAs from the same family are expected to be co-regulated [21, 22]. Consequently, adding additional co-regulated reference genes would not significantly improve the combined value of the genes as normalizers. The optimal number of reference miRNAs to use was then calculated using the pairwise variation measure *V* as described by Vandesompele et al*.* [21], and Chang et al*.* [11]. *V* = 0.15 has been suggested as a suitable threshold below which the inclusion of additional reference miRNAs is not required, i.e. addition of further reference miRNAs will not significantly improve stability [21].

The performance of previously identified candidate prognostic miRNAs for each cancer type [16, 18] was tested by analyzing the association between OS and expression of the candidate prognostic miRNAs using both global mean normalization and normalization with the identified reference miRNAs.

#### Effect of sample age (CRC cohort and PC cohort)

Global mean miRNA expression was plotted for each sample according to the age of the tumor block, and the effect of age was estimated using linear regression with age as a continuous variable.

#### Methodological samples

Correlations between related samples were described using correlation coefficients, *r*_*s*_. Linear mixed effects models with miRNA and samples as random effects were used to quantify technical variability corresponding to the between-sample and within-sample variation and were reported as standard deviations (SD) in C_q_. SDs after normalization with global mean [10], the mean of the optimal group of miRNAs, or the mean of the two best reference miRNAs were also calculated. Hierarchical clustering was performed using the miRNAs measured on all samples. Euclidean distance and complete linkage were used with four different normalization strategies: raw values (no normalization), global mean, mean of optimal reference miRNAs, and mean of the two best reference miRNAs. Additionally, 1-Pearson correlation was used as distance metric. Dendrograms were made for each of the mentioned clustering procedures. Clustering was performed using the function *hclust* in the *stats* package in R, and clusters were defined by *cutreeDynamicTree* in the package *dynamic-TreeCut* [23]. *P* < 0.05 was considered statistically significant. The statistical software package R [24] version 3.0.2 (www.r-project.org) was used for all analyses. An overview of the sample cohorts and associated analyses is presented in Figure S1 in Additional file 1.

### Ethics

The study was approved by the Regional Scientific Ethics Committee of the Capital Region of Denmark (http://www.regionh.dk/vek, approval numbers: H-KA-20060181 and H-1-2010-081). The CRC study was a retrospective study wherein the ethics committee waived the requirement for obtaining informed consent. The PC study included participants prospectively and all participants signed an informed consent form allowing for publication of the results.

## Results

### Quality assessment

The median RNA yield from the purification of FFPE and frozen samples were 3.3 μg and 33.4 μg, respectively. Seventy-four clinical samples (6 CRC and 68 PC samples) had a 260/280-ratio <1.8, leaving 197 CRC samples and 188 PC samples for further analyses. After first removing all C_q_ values above 32 and then removing miRNAs with more than 5 % undetermined measurements, 199 miRNAs (CRC) and 179 miRNAs (PC) were left for reference miRNA- and sample age analysis. Five methodological samples were excluded based on 260/280 nm or 260/230 nm ratio and an additional frozen sample was excluded because no comparison FFPE blocks were left (Table S1 in Additional file 1). Corresponding raw miRNA expression data, sample age, and survival data are provided in Datasets S1–S3 in Additional file 2, Additional file 3, and Additional file 4, respectively.

### Identification of reference miRNAs

The 20 miRNAs that correlated best with global mean miRNA expression within each cancer type are listed in Table S2 and S3 in Additional file 1, ordered by their stability score. A few of the miRNAs were associated with OS, but the hazard ratio (HR) estimates for these miRNAs were close to the HR estimate of global mean miRNA expression. Even though the identified reference miRNAs differed between cancer types, nine miRNAs were identified in both cancer types, namely: miR-24-3p, miR-26a-5p, miR-27a-3p, miR-28-5p, miR-103a-3p, miR-106b-5p, miR-152-3p, miR-199a-3p, and miR-374a-5p.

The following miRNAs were not considered in the calculation of the optimal number of miRNAs because they were in the same miRNAs family as other miRNAs with better stability scores: miR-30c-5p and miR-28-5p in CRC, and miR-26a-5p, miR-130a-3p, miR-374a-5p, and let-7f-5p in PC. The optimal number of reference miRNAs to use was 4 in CRC and 10 in PC (Table 1 and Table 2). MiR-27a-3p and miR-103a-3p were included in the optimal number of reference miRNAs in both cancer types. Four reference miRNAs were adequate to reach the suggested cut-off of V < 0.15 in both types of cancer samples.

**Table 1:** Optimal number of reference miRNAs – colorectal cancer

Number of miRNAs	V^a^	ΔV^a^	miRNAs added	Stability increased?
2	–	–	**miR-103a-3p**, **miR-152-3p**	–
3 vs 2	0.3475	–	**miR-132-3p**	–
4 vs 3	0.1400	-0.2075	**miR-27a-3p**	yes
5 vs 4	0.1728	0.0328	miR-140-5p	no
6 vs 5	0.1145	-0.0583	miR-30b-5p	yes
7 vs 6	0.1189	0.0044	miR-339-5p	no
8 vs 7	0.1005	-0.0185	miR-331-3p	yes
9 vs 8	0.0991	-0.0014	miR-374a-5p	yes
10 vs 9	0.0778	-0.0212	miR-652-3p	yes
11 vs 10	0.0689	-0.0089	miR-335-5p	yes
12 vs 11	0.0728	0.0040	miR-185-5p	no
13 vs 12	0.0588	-0.0140	miR-151-5p	yes
14 vs 13	0.0614	0.0025	miR-106b-5p	no
15 vs 14	0.0503	-0.0111	miR-199a-3p	yes
16 vs 15	0.0574	0.0071	miR-425-5p	no
17 vs 16	0.0426	-0.0147	miR-26a-5p	yes
18 vs 17	0.0685	0.0259	miR-24-3p	no

MiRNAs in bold are the ones that are included in the optimal number of references to use.

^a^The stability measure V is calculated according to Vandesompele et al. [21]

**Table 2:** Optimal number of reference miRNAs – pancreatic cancer

Number of miRNAs	V^a^	ΔV^a^	miRNAs added	Stability increased?
2	–	–	**miR-103a-3p**, **miR-374b-5p**	–
3 vs 2	0.1884	–	**miR-361-5p**	–
4 vs 3	0.1328	-0.0556	**let-7 g-5p**	yes
5 vs 4	0.1141	-0.0186	**miR-28-5p**	yes
6 vs 5	0.0962	-0.0179	**miR-29a-3p**	yes
7 vs 6	0.0831	-0.0131	**miR-301a-3p**	yes
8 vs 7	0.0631	-0.0200	**miR-340-5p**	yes
9 vs 8	0.0596	-0.0036	**miR-27a-3p**	yes
10 vs 9	0.0522	-0.0073	**miR-106b-5p**	yes
11 vs 10	0.0527	0.0005	miR-152-3p	no
12 vs 11	0.0549	0.0021	miR-26b-5p	no
13 vs 12	0.0464	-0.0084	miR-660-5p	yes
14 vs 13	0.0470	0.0006	miR-24-3p	no
15 vs 14	0.0517	0.0047	miR-23b-3p	no
16 vs 15	0.0569	0.0052	miR-199a-3p	no

MiRNAs in bold are the ones that are included in the optimal number of references to use.

^a^ The stability measure V is calculated according to Vandesompele *et al.* [21]

The ratio of CRC reference miRNA expression (*n* = 4) to global mean miRNA expression was independent of tumor cell percentage (Figure S2 in Additional file 1). The correlation between the top four PC reference miRNAs and global mean miRNA expression was similar in tissue samples from PC, normal pancreas, and chronic pancreatitis (Figure S3 in Additional file 1). Moreover, when we used the same methodology as mentioned above to identify reference miRNAs in samples from normal pancreas (*n* = 20) or chronic pancreatitis (*n* = 20) alone, many of the same candidate reference miRNAs as in the PC cohort were found (Table S4 and S5 in Additional file 1). The estimated hazard ratios for previously identified candidate prognostic miRNAs [16, 18] were comparable for global mean normalization and normalization using the identified reference miRNAs (Table S6 in Additional file 1).

### Effect of sample age

**Fig. 1:**
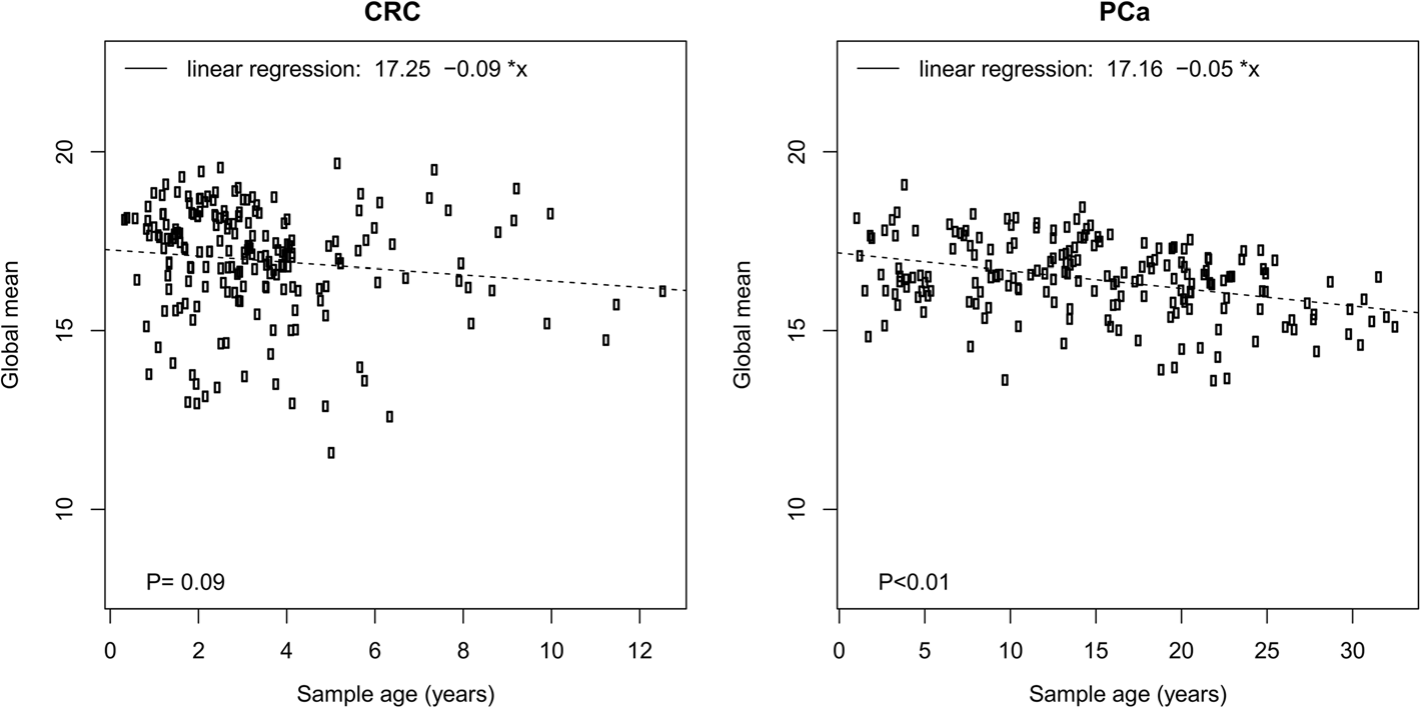
Association between sample age and global mean miRNA expression for CRC (left) and PC (right) samples. Age of the tumor blocks are plotted on the x-axis and global mean miRNA expression in 40-C_q_ values on the y-axis. A linear regression line has been plotted and the formula is shown.

There was a trend toward lower global mean miRNA expression with increasing sample age in both tumor types (Fig. 1), but this effect was only significant in PC samples. CRC samples were up to 12 years old, but most were 0–5 years old. The ages of PC samples were more evenly distributed and ranged from 0 to more than 30 years. The effect of age in PC samples was -0.05 C_q_/year and highly significant (*p* < 0.01). After global mean normalization, only two miRNAs showed a significant effect of storage time on expression in both cancer types. These were miR-197-3p: -0.09 C_q_/year (*p* = 0.02) in CRC and -0.04 C_q_/year (*p* < 0.01) in PC; and miR-425-5p: -0.05 C_q_/year (*p* = 0.03) in CRC and -0.02 C_q_/year (*p* < 0.01) in PC.

### Sources of variability

**Fig. 2:**
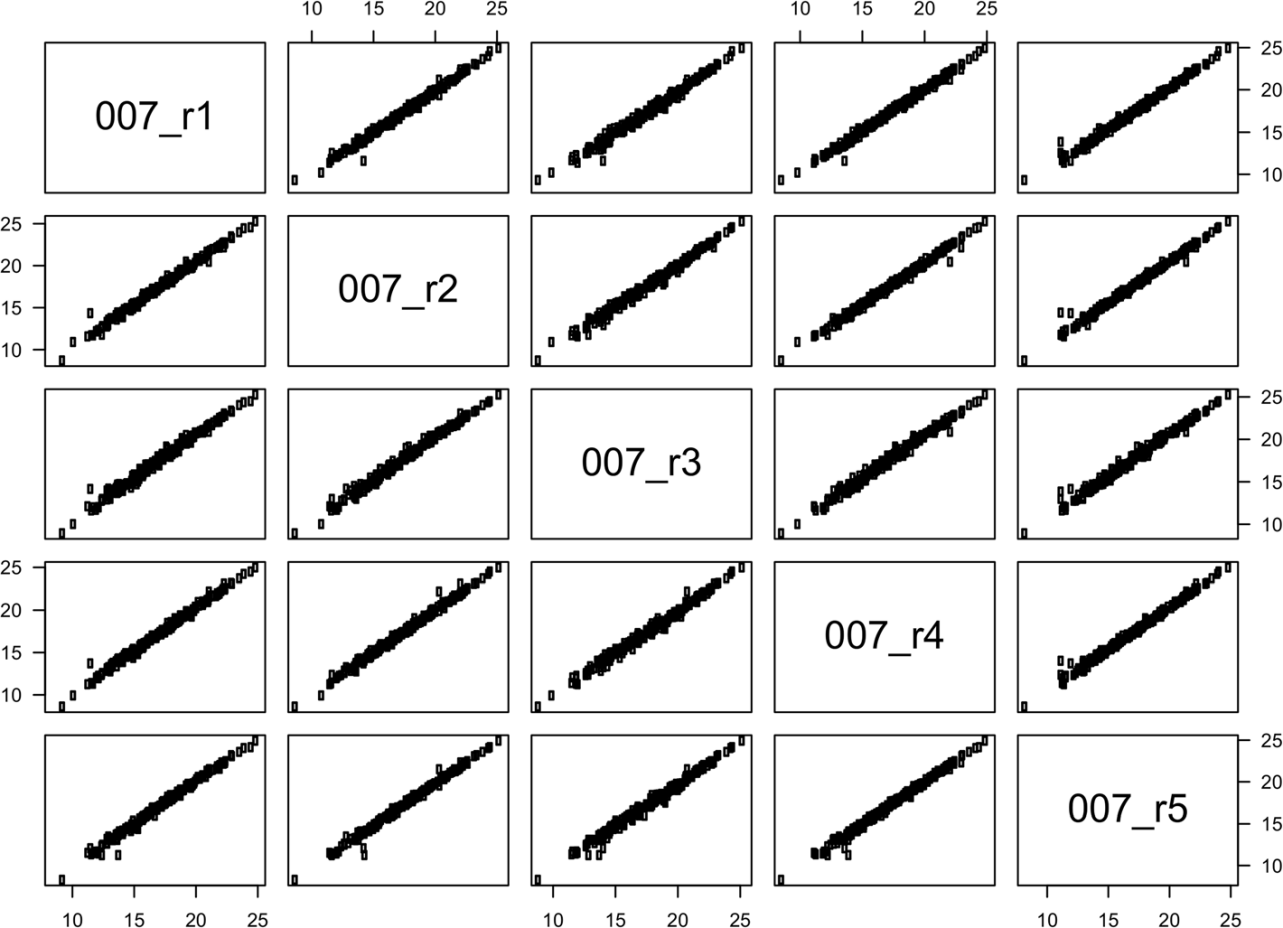
Correlation between miRNA expression profiles measured in five runs from the same purification. Correlations are illustrated by a pairs plot with axes showing miRNA expression in 40-C_q_ values. The sample numbers correspond to the project IDs in Table S1 in Additional file 1.

**Fig. 3:**
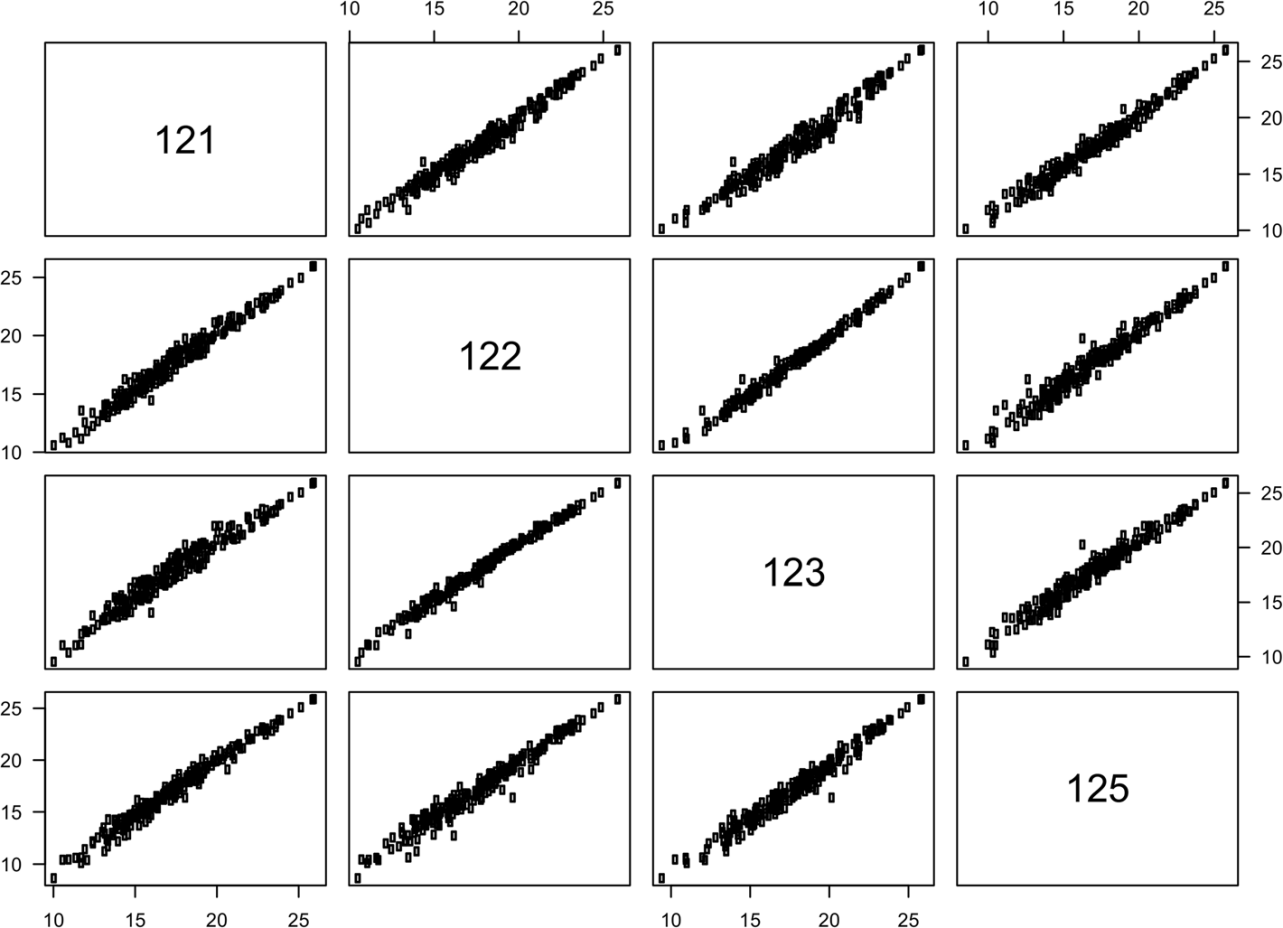
Correlation between miRNA expression profiles measured in four samples from the same tumor block. Correlations are illustrated by a pairs plot with axes showing miRNA expression in 40-C_q_ values. The sample numbers correspond to the project IDs in Table S1 in Additional file 1.

Two hundred miRNAs were determined in at least 95 % of the methodological samples. Measurements of miRNA expression in five samples from the same cDNA reaction ("technical replicates") were highly correlated: *r*_*s*_ = 0.993–0.996 (Table 3 and Fig. 2). The total variation in SD in a miRNA expression measurement was 0.31 C_q_ with equal contributions from within- and between-sample variation. MiRNA expression in the five samples sectioned from adjacent areas of the same tumor block ("biological replicates") also showed a strong correlation: *r*_*s*_ = 0.98–0.99 (Fig. 3). The SD on a miRNA expression measurement was 0.52 C_q_ for the biological replicates. The correlations between the three other pairs of biological replicates were 0.99, 0.93, and 0.99. This should be compared to the median correlation between unrelated samples, i.e. the inter-individual correlation, which was 0.88. Global mean normalization lowered the SD to 0.24 C_q_ (technical replicates) and 0.42 C_q_ (biological replicates). Paraffin embedding and formalin fixation lowered the global mean miRNA expression. The effect was smaller for 2-day fixation: -0.50 C_q_, than for 3- and 6-day fixation: -1.56 C_q_ and -1.14 C_q_, respectively. This corresponds to a reduction of 30–65 % assuming 100 % PCR efficiency. The variability between the frozen and differently formalin-fixed samples was much greater than the variability observed for the biological replicates taken from the same FFPE block, but the mean correlation was still high (Table 3). With the exception of the comparison between technical replicates, using the two best reference miRNAs (miR-103a-3p and miR-152-3p) or the previously identified optimal number of reference miRNAs (*n* = 4) for normalization also tended to lower the SD, although to a lesser degree than with global mean normalization (Table 3).

**Table 3:** Variability in miRNA expression measurements: technical replicates, biological replicates, and frozen versus FFPE tissue

			Variability, SD (C_q_)					
		Correlation^a^, *r*_*s*_	Raw data			Total variability with normalized data		
	Samples	median (range)	Individual miRNA	Inter-sample	Total	Global mean	Optimal number of refs. (*n* = 4)	Two best refs.
Technical replicates	5	0.995 (0.993–0.996)	0.24	0.19	0.31	0.24	0.32	0.40
Biological replicates	4	0.985 (0.98–0.99)^b^	0.42	0.30	0.52	0.42	0.46	0.49
Frozen vs. FFPE	17^c^	0.92 (0.66–0.96)	1.35	1.00	1.68	1.18	1.35	1.31

^a^ For frozen versus FFPE, the correlations were calculated between formalin-fixed and corresponding frozen samples (12 total correlations).

^b^ The median correlation between unrelated biological replicates, i.e. the inter-individual correlation, was 0.88 (range 0.84–0.91).

^c^ Sample distribution: 5 frozen, 4 x 2-day fixation, 5 x 3-day fixation, and 3 x 6-day fixation.

Abbreviations: FFPE, formalin-fixed paraffin-embedded; SD, standard deviation; C_q_, quantification cycle; *r*_*s*_, Spearman's rank correlation coefficient; refs., references.

### Hierarchical clustering

One hundred and seventy-four miRNAs were determined in all the 31 methodological samples and only these miRNAs were used for clustering. Clustering dendrograms are shown in Figures S4–S8 in Additional file 1. Using 1-Pearson correlation as a distance metric, 87 % of samples clustered together with at least one other sample from the same tumor. Using Euclidean distance on the raw expression data, 74 % of samples clustered with at least one other sample from the same tumor. This percentage improved numerically with normalization using global mean, optimal number of reference miRNAs (*n* = 4), and the two best reference miRNAs (miR-103a-3p and miR-152-3p) to 90 %, 81 %, and 84 %, respectively. Two pairs of biological replicates did not cluster together when raw data was used, but did so in all of the normalized data dendrograms. Frozen samples from different tumors tended to cluster together.

### Repeat analysis using all measurements

To address the potential bias introduced by removing low expression measurements, we repeated all analyses without removal of any measurements. The addition of low expression measurements resulted in an increased variability between replicates that could not be lowered by normalization (Table S7 in Additional file 1). The small decrease in global mean miRNA expression with increasing sample age was still present (CRC: -0.07 C_q_/year, *p* = 0.03; PC: -0.02 C_q_/year, *p* < 0.01). There was a high correlation between high-quality means (measurements with C_q_ > 32 and miRNAs with >5 % undetermined removed) and low-quality means (no measurements removed) (Figure S9 in Additional file 1). The list of candidate reference miRNAs identified using all measurements was similar to the list identified using high quality measurements (Additional file 1: Table S8 and S9). Of note, miR-103a-3p was still the most stable or third most stable reference miRNA.

## Discussion

MiRNAs hold great promise as biomarkers for the diagnosis, prognosis, and prediction of treatment benefit in patients with cancer, and they are also potential targets for new cancer drugs. In order to utilize miRNAs for these purposes, it is paramount to first learn about the precise dysregulation of individual miRNAs in various disease states. To this end, optimal normalization is essential. In whole “miRNome” studies, global mean normalization is the established standard. But in studies with fewer miRNAs, normalization with stable global mean-associated reference miRNAs is the best option [10–15].

To the best of our knowledge, we have presented in this study the first unbiased identification of stable miRNA reference genes in FFPE cancer tissue. This is also the first study to identify miRNA reference genes in PC tissue, and it is the largest study of its kind to date. Normalization with the selected CRC reference miRNAs was able to slightly reduce variability between biological replicates and between frozen and FFPE samples, and it improved hierarchical clustering results. The reason why the reference miRNAs did not decrease variability for technical replicates could be that the variability in the raw data was very low. As shown in Table S4 in Additional file 1, 10 out of the 20 candidate reference miRNAs for CRC tissue and 8 out of the 20 candidate reference miRNAs for PC tissue have been identified as stable global mean-associated reference miRNAs in previous studies on frozen samples. In fact, at least one reference miRNA from each of the previously reported studies was represented in our top-20 lists (Table S4 in Additional file 1). This indicates that some reference miRNAs are suitable for normalization of both frozen- and FFPE samples, which has been suggested previously [12].

Reference miRNAs are not necessarily equally suited for normalization of miRNA expression in cancer cells and in normal cells. However, the expression of the identified CRC reference miRNAs was independent of tumor cell content. Moreover, the expression of the identified PC reference miRNAs was strongly correlated with global mean miRNA expression in both cancer and non-cancer samples. Therefore, these reference miRNAs could be suitable for normalization regardless of the ratio of tumor- versus normal cells in the sample. Further, several of the candidate reference miRNAs we identified have previously been shown to be stably expressed in normal tissue from multiple organs [10, 12, 14]. The most stable global mean-associated miRNA in both of our cohorts was miR-103a-3p. This miRNA has also been identified as a stable normalizer in frozen kidney and lung cancer samples [12, 13]. Interestingly, in the study by Peltier et al. [12], the authors found that miR-103a-3p was only the fourth most stable reference miRNA in frozen lung cancer tissue, while it was the most stable reference miRNA in FFPE lung cancer tissue. Thus, this miRNA could be especially suitable for normalization in FFPE cancer tissue. In the same study, miR-16 – a commonly used reference miRNA – was the least stable of seven candidate miRNAs in both frozen and FFPE lung cancer samples. MiR-16 was not identified as a candidate reference miRNA in our study, and its relevance as a normalizer in FFPE cancer tissue is uncertain.

We identified a significant but modestly sized decrease in miRNA expression as a function of storage time in PC. The trend was similar in CRC samples, but it was not significant. The effect in PC amounts to a 50 % reduction of the miRNA expression over 20 years. Siebolts et al. found a very similar rate of decline of miR-16 expression in FFPE blocks stored for up to 27 years [25]. An effect of this size should not have a detrimental impact on the use of FFPE samples for clinical biomarkers in PC or CRC, because these samples are often only a few months to a couple of years old when used. Also, normalization would mitigate some of the difference, as demonstrated in two other studies in which the authors did not see any effect of storage time in miRNA expression normalized to *RNU6B* or to other miRNAs [26, 27].

An excellent reproducibility of the chosen platform was demonstrated with highly correlated miRNA expression in technical replicates. The biological replicates were different sections from the same tumor block. The variability in these replicates is the sum of biological differences in the tumor block and variability in sectioning, purification, reverse transcription, and amplification. Even with all these additional sources of variability, we found an excellent correlation between these samples, with only a minor increase in SD, from 0.31 to 0.52 C_q_, compared to the technical variability caused by the amplification process alone. This suggests that miRNA expression is not very heterogeneous within an FFPE tumor block.

Formalin fixation and paraffin embedding caused a decline in miRNA expression of between 0.5 and ~1.5 C_q_; yet, correlation between miRNA expression in frozen- and FFPE tissue from the same tumor was still high. Hoefig et al. also found a 1.0–1.5 C_q_ decline in miRNA expression in formalin-fixed compared to frozen liver- and tonsil samples [28]. The decrease in global mean miRNA expression could be a result of miRNA degradation, excessive fixation of miRNA in the FFPE tissue with suboptimal purification, or small fragmented ribosomal- and messenger RNA interfering with the miRNA signal on the array. Many previous studies have reported a good correlation between frozen- and FFPE samples [28–32]. The variability between frozen and FFPE samples was high in our study, and frozen samples from different tumors tended to cluster together in the hierarchical clustering analysis. Moreover, global mean normalization did not greatly improve the clustering of frozen samples. These findings could be a result of formalin-fixation causing non-uniform changes in miRNA measurability. They highlight the importance of doing large scale studies in both frozen and FFPE samples and not relying on direct portability of results between the two.

The quality of the purified RNA was lower in the PC cohort than in the CRC cohort, resulting in a higher proportion of excluded samples in the PC cohort. The PC samples were purified and analyzed in smaller batches distributed over a longer period compared to the CRC samples, which could have decreased overall quality of this sample cohort. In addition, the purification kit used for the PC cohort, which differed from the kit used in the CRC cohort, could have been less effective. Finally, the samples in the PC cohort were older which could also have influenced RNA quality.

## Conclusions

Low expression measurements (C_q_ > 32) were removed before undertaking the analyses. This was done to reduce noise in the data, but it could also introduce a bias to the results. Therefore, all of the analyses were repeated without removal of low expression measurements, but this did not result in any major changes in the results, apart from the anticipated increase in variability. It is also important to note that several different technologies are used for miRNA expression quantification apart from RT-qPCR, e.g. hybridization-based arrays and sequencing technologies [33], and our findings may not be applicable to all such technologies. In summary, we have identified stable global mean-associated reference miRNAs for use in miRNA expression studies on FFPE cancer tissue from patients with colorectal and pancreatic cancer. This is the first study to search specifically for reference miRNAs for use in FFPE cancer tissue. We also found that the length of storage is not a significant determinant of miRNA abundance in FFPE cancer tissue and that intra-block miRNA expression heterogeneity seems to be low. Formalin fixation caused a decline in miRNA expression, but expression profiles from frozen and FFPE samples from the same tumor were generally still highly correlated. These results should provide valuable information for the planning and execution of future miRNA biomarker studies in patients with cancer.

### Availability of data and materials

All source data can be found in the additional supporting files.

## Additional files

Additional file 1:
**Supplementary tables, figures, and references.** (PDF) (PDF 869 kb) Additional file 2:
**Dataset S1: MiRNA expression in colorectal cancer samples.** (Microsoft Excel spreadsheet) (XLSX 412 kb) Additional file 3:
**Dataset S2. MiRNA expression in pancreatic cancer samples.** (Microsoft Excel spreadsheet) (XLSX 355 kb) Additional file 4:
**Dataset S3. MiRNA expression in methodological samples.** (Microsoft Excel spreadsheet) (XLSX 75 kb)

## Abbreviations

CPH: Cox proportional hazards model
C_q_: quantification cycle
CRC: colorectal cancer
FFPE: formalin-fixed paraffin-embedded
HE: hematoxylin and eosin
HR: hazard ratio
mCRC: metastatic CRC
miRNA: microRNA
MRE: miRNA recognition element
mRNA: messenger RNA
OS: overall survival
PC: pancreatic cancer
RISC: RNA-induced silencing complex
RT: reverse transcribed/reverse transcription
RT-qPCR: reverse transcription quantitative polymerase chain reaction
SD: standard deviation
