# Supplementary material for: MicroRNA Expression in Formalin-fixed Paraffin-embedded Cancer Tissue: Identifying Reference MicroRNAs and Variability
Source: BMC Cancer. 2015 Dec 29;15:1024. doi: 10.1186/s12885-015-2030-2 (PMC4696166; doi:10.1186/s12885-015-2030-2)
Supplement: Additional file 1: — Supplementary tables, figures, and references. (PDF) (PDF 869 kb) [file 12885_2015_2030_MOESM1_ESM.pdf]

SUPPLEMENTARY INFORMATION

Supplementary Table S1. Methodological samples

Project ID	Tumor number	Block type	Description	Excluded[a]
2	1	FFPE	First cut	
92	1	FFPE	Second cut	
57	2	FFPE	First cut	
97	2	FFPE	Second cut	
166	3	FFPE	First cut	
245	3	FFPE	Second cut	
120	4	FROZEN	Fresh frozen tumor block	
121	4	FFPE1	2-day fixation first cut	
122	4	FFPE1	2-day fixation second cut	
123	4	FFPE1	2-day fixation third cut	
124	4	FFPE1	2-day fixation fourth cut	x
125	4	FFPE1	2-day fixation fifth cut	
126	4	FFPE2	3-day fixation	
127	4	FFPE3	6-day fixation	x
136	5	FROZEN	Fresh frozen tumor block	
137	5	FFPE1	2-day fixation	
138	5	FFPE2	3-day fixation	
139	5	FFPE3	6-day fixation	
6	6	FROZEN	Fresh frozen tumor block	
7	6	FFPE1	2-day fixation	
7r1	6	FFPE1	2-day fixation first run	
7r2	6	FFPE1	2-day fixation second run	
7r3	6	FFPE1	2-day fixation third run	
7r4	6	FFPE1	2-day fixation fourth run	
7r5	6	FFPE1	2-day fixation fifth run	
8	6	FFPE2	3-day fixation	
9	6	FFPE3	6-day fixation	
10	7	FROZEN	Fresh frozen tumor block	
11	7	FFPE1	2-day fixation	
12	7	FFPE2	3-day fixation	
13	7	FFPE3	6-day fixation	
256	8	FROZEN	Fresh frozen tumor block	
257	8	FFPE1	2-day fixation	x
258	8	FFPE2	3-day fixation	
259	9	FROZEN	Fresh frozen tumor block	(x)[b]
260	9	FFPE1	2-day fixation	x
261	9	FFPE2	6-day fixation	x

[a] Excluded based on 260/280 nm or 260/230 nm ratio.

[b] Project ID 259 was not used because no formalin-fixed sample from that tumor was usable.

Supplementary Table S2. Reference miRNAs – colorectal cancer

MiRNA name[a]	Correlation to global mean C_q r_s[b]	Correlation to OS[c] HR/C_q	 *P*	Stability[d] M score
Global mean	–	0.933	0.215	–
hsa-miR-103a-3p	0.891	0.950	0.220	1.044
hsa-miR-152-3p	0.914	0.953	0.257	1.074
hsa-miR-132-3p	0.880	0.961	0.317	1.094
hsa-miR-27a-3p	0.884	0.940	0.129	1.099
hsa-miR-140-5p	0.910	0.924	0.037	1.107
hsa-miR-30b-5p	0.932	0.966	0.282	1.118
hsa-miR-339-5p	0.887	0.952	0.277	1.122
hsa-miR-331-3p	0.906	0.964	0.397	1.131
hsa-miR-374a-5p	0.893	0.964	0.321	1.142
hsa-miR-652-3p	0.886	0.954	0.290	1.152
hsa-miR-335-5p	0.882	0.933	0.091	1.171
hsa-miR-185-5p	0.902	0.942	0.241	1.179
hsa-miR-30c-5p	0.930	0.961	0.217	1.186
hsa-miR-151a-5p	0.901	0.974	0.519	1.213
hsa-miR-106b-5p	0.886	0.964	0.332	1.215
hsa-miR-199a-3p	0.904	0.970	0.374	1.215
hsa-miR-28-5p	0.892	0.960	0.251	1.226
hsa-miR-425-5p	0.901	1.002	0.956	1.246
hsa-miR-26a-5p	0.912	0.967	0.289	1.293
hsa-miR-24-3p	0.892	0.982	0.613	1.681

[a] MiRBase version 21 (www.mirbase.org, accessed July 25 2014).

[b] Spearman's rank correlation coefficient.

[c] Correlation to OS duration tested using Cox proportional hazards model.

[d] Stability score calculated according to the method described in Vandesompele et al. [1]

Abbreviations: OS, overall survival; C_q, quantification cycle; HR/C_q, hazard ratio per 40-C_q increase.

Supplementary Table S3. Reference miRNAs – pancreatic cancer

	Correlation to global mean C_q	Correlation to OS[c]		Stability[d]
**MiRNA name[a]**	r_s[b]	**HR/C_q**	P	**M score**
Global mean	–	1.101	0.249	–
hsa-miR-103a-3p	0.943	1.058	0.309	0.670
hsa-miR-374b-5p	0.942	1.079	0.201	0.680
hsa-miR-361-5p	0.918	1.068	0.288	0.749
hsa-miR-374a-5p	0.911	1.058	0.356	0.754
hsa-let-7g-5p	0.917	1.072	0.197	0.756
hsa-miR-28-5p	0.924	1.082	0.198	0.761
hsa-miR-29a-3p	0.913	1.093	0.245	0.783
hsa-miR-301a-3p	0.921	1.037	0.480	0.786
hsa-miR-340-5p	0.950	1.103	0.148	0.791
hsa-miR-27a-3p	0.923	1.146	0.043	0.792
hsa-miR-106b-5p	0.885	1.039	0.554	0.810
hsa-miR-152-3p	0.914	1.088	0.169	0.821
hsa-miR-26b-5p	0.915	1.050	0.262	0.829
hsa-miR-660-5p	0.902	1.013	0.843	0.837
hsa-miR-26a-5p	0.909	1.064	0.167	0.843
hsa-miR-24-3p	0.895	1.088	0.087	0.850
hsa-miR-130a-3p	0.884	1.074	0.117	0.911
hsa-miR-23b-3p	0.885	1.034	0.524	0.922
hsa-let-7f-5p	0.887	1.082	0.084	0.948
hsa-miR-199a-3p	0.897	1.082	0.064	0.991

[a] MiRBase version 21 (www.mirbase.org, accessed July 25 2014).

[b] Spearman's rank correlation coefficient.

[c] Correlation to OS duration tested using Cox proportional hazards model.

[d] Stability score calculated according to the method described in Vandesompele et al. [1]

Abbreviations: OS, overall survival; C_q, quantification cycle; HR/C_q, hazard ratio per 40-C_q increase.

Supplementary Table S4. Reference miRNAs – normal pancreas (n=20)

MiRNA name[a]	Correlation to global mean C_q r_s[b]	Stability[c] M score	Also in pancreatic cancer top 20 list
hsa-miR-374a-5p	0.880	0.284	x
hsa-miR-660-5p	0.886	0.308	x
hsa-miR-152-3p	0.895	0.322	x
hsa-miR-361-5p	0.856	0.328	x
hsa-miR-29b-3p	0.848	0.329	
hsa-miR-101-3p	0.871	0.333	
hsa-miR-93-5p	0.871	0.333	
hsa-mir-331-3p	0.874	0.333	
hsa-miR-16-5p	0.847	0.336	
hsa-mir-125b	0.886	0.338	
hsa-miR-27a-3p	0.908	0.338	x
hsa-miR-340-5p	0.853	0.339	x
hsa-miR-19a-3p	0.877	0.340	
hsa-miR-103a-3p	0.871	0.340	x
hsa-miR-20b-5p	0.857	0.341	
hsa-miR-126-3p	0.877	0.345	
hsa-miR-301a-3p	0.854	0.358	x
hsa-miR-221-3p	0.901	0.359	
hsa-miR-454-3p	0.851	0.360	
hsa-miR-15a-5p	0.884	0.384	

[a] MiRBase version 21 (www.mirbase.org, accessed September 23 2014).

[b] Spearman's rank correlation coefficient.

[c] Stability score calculated according to the method described in Vandesompele et al. [1]

Abbreviations: C_q, quantification cycle.

Supplementary Table S5. Reference miRNAs – chronic pancreatitis (n=20)

MiRNA name[a]	Correlation to global mean C_q r_s[b]	Stability[c] M score	Also in pancreatic cancer top 20 list
hsa-miR-191-5p	0.976	0.421	
hsa-miR-374a-5p	0.964	0.437	x
hsa-miR-106b-5p	0.956	0.450	x
hsa-miR-301a-3p	0.979	0.455	x
hsa-miR-340-5p	0.953	0.464	x
hsa-miR-454-3p	0.983	0.469	
hsa-miR-99b-5p	0.977	0.477	
hsa-miR-744-5p	0.961	0.480	
hsa-miR-361-5p	0.979	0.481	x
hsa-miR-24-3p	0.962	0.487	x
hsa-miR-101-3p	0.986	0.499	
hsa-miR-374b-5p	0.968	0.540	x
hsa-miR-103a-3p	0.967	0.541	x
hsa-miR-126-3p	0.965	0.558	
hsa-miR-128-3p	0.962	0.563	
hsa-miR-29a-3p	0.956	0.571	x
hsa-miR-193a-3p	0.946	0.576	
hsa-miR-660-5p	0.956	0.595	x
hsa-miR-130a-3p	0.946	0.718	x
hsa-miR-410-3p	0.971	0.767	

[a] MiRBase version 21 (www.mirbase.org, accessed September 23 2014).

[b] Spearman's rank correlation coefficient.

[c] Stability score calculated according to the method described in Vandesompele et al. [1]

Abbreviations: C_q, quantification cycle.

Supplementary Table S6. Association between expression of selected miRNAs and overall survival using different normalizations

Pancreatic cancer

miRNA[a]	Normalization method[b]	HR[c]	95% CI	p
hsa-miR-212-3p	Global mean	1.26	1.06–1.49	<0.01
hsa-miR-212-3p	Mean of reference miRNAs	1.16	0.99–1.37	0.07
hsa-miR-675-5p	Global mean	1.24	1.00–1.55	0.05
hsa-miR-675-5p	Mean of reference miRNAs	1.17	0.96–1.43	0.13
hsa-miR-187-3p	Global mean	0.75	0.59–0.95	0.02
hsa-miR-187-3p	Mean of reference miRNAs	0.74	0.59–0.94	0.01

Colorectal cancer

miRNA[a]	Normalization method[b]	HR[c]	95% CI	p
hsa-miR-592	Global mean	0.86	0.71–1.06	0.16
hsa-miR-592	Mean of reference miRNAs	0.91	0.75–1.10	0.32
hsa-miR-29b-3p	Global mean	0.83	0.68–1.00	0.05
hsa-miR-29b-3p	Mean of reference miRNAs	0.88	0.70–1.09	0.23
hsa-miR-196b-5p	Global mean	0.96	0.82–1.14	0.66
hsa-miR-196b-5p	Mean of reference miRNAs	1.04	0.85–1.26	0.72

The miRNAs were selected from the list of candidate prognostic miRNAs in two previously published papers [2, 3]. Since we used all data points without any adjustments for these analyses, results will differ from the ones reported in the previous papers.

[a] MiRBase version 21 (www.mirbase.org, accessed September 23 2014).

[b] The identified optimal number of reference miRNAs were used for each cancer type (from Table 1 and 2)

[c] Overall survival HR per inter-quartile range increase in 40-C_q, calculated using Cox proportional hazard model.

Abbreviations: HR, hazard ratio; CI, confidence interval.

Supplementary Table S7. Variability in miRNA expression measurements (without removal of low expression values)

			**Variability, SD (C_q)**					
		**Correlation[a], r_s**	**Raw data**			**Total variability with normalized data**		
	**Samples**	**median (range)**	**Individual miRNA**	**Inter-sample**	**Total**	**Global mean**	**Optimal number of refs. (*n*=4)**	**Two best refs.**
Technical replicates	5	0.975 (0.967–0.979)	0.85	0.24	0.88	0.85	0.86	0.87
Biological replicates	4	0.965 (0.95–0.97)	0.95	0.18	0.97	0.96	1.00	1.02
Frozen vs. FFPE	17[b]	0.92 (0.77–0.96)	1.95	0.98	2.19	1.89	1.95	1.94

[a] For frozen versus FFPE, the correlations were calculated between formalin-fixed- and corresponding frozen samples (12 total correlations).

[b] Sample distribution: 5 frozen, 4 x 2-day fixation, 5 x 3-day fixation, and 3 x 6-day fixation.

Abbreviations: FFPE, formalin-fixed paraffin-embedded; SD, standard deviation; C_q, quantification cycle; r_s, Spearman's rank correlation coefficient; refs., references.

Supplementary Table S8. Reference miRNAs – colorectal cancer (using all measurements)

	Correlation to global mean C_q	Correlation to OS[c]		Stability[d]
**MiRNA name[a]**	r_s[b]	HR/C_q	P	**M score**
Global mean	–	0.904	0.237	–
hsa-miR-335-5p	0.908	0.963	0.254	1.461
hsa-miR-152-3p	0.888	0.984	0.727	1.467
hsa-miR-103a-3p	0.880	0.964	0.377	1.477
hsa-miR-30b-5p	0.899	0.977	0.490	1.495
hsa-miR-15b-5p	0.873	1.023	0.356	1.530
hsa-miR-425-5p	0.903	0.977	0.603	1.534
hsa-miR-652-3p	0.889	0.967	0.458	1.545
hsa-miR-376a-3p	0.881	0.986	0.786	1.552
hsa-miR-132-3p	0.874	0.988	0.750	1.553
hsa-miR-151a-5p	0.921	0.974	0.410	1.575
hsa-miR-331-3p	0.877	0.981	0.704	1.577
hsa-miR-93-3p	0.873	1.029	0.425	1.590
hsa-miR-26a-5p	0.882	0.981	0.583	1.662
hsa-miR-106b-5p	0.880	1.016	0.724	1.695
hsa-miR-7-1-3p	0.875	0.994	0.874	1.707
hsa-miR-130b-3p	0.878	0.999	0.972	1.734
hsa-miR-30c-5p	0.898	0.972	0.366	1.755
hsa-miR-24-3p	0.888	0.938	0.320	1.796
hsa-miR-218-5p	0.887	0.962	0.244	1.835
hsa-miR-15b-3p	0.873	1.008	0.613	2.398

[a] MiRBase version 21 (www.mirbase.org, accessed July 25 2014).

[b] Spearman's rank correlation coefficient.

[c] Correlation to OS duration tested using Cox proportional hazards model.

[d] Stability score calculated according to the method described in Vandesompele et al. [1]

Abbreviations: OS, overall survival; C_q, quantification cycle; HR/C_q, hazard ratio per 40-C_q increase.

Supplementary Table S9. Reference miRNAs – pancreatic cancer (using all measurements)

	**Correlation to global mean C_q**	**Correlation to OS**[c]		**Stability**[d]
**MiRNA name**[a]	r_s[b]	**HR/C_q**	P	**M score**
Global mean	–	1.210	0.145	–
hsa-miR-103a-3p	0.906	1.103	0.064	0.681
hsa-miR-374b-5p	0.892	1.129	0.033	0.715
hsa-let-7g-5p	0.877	1.094	0.034	0.761
hsa-miR-361-5p	0.885	1.113	0.097	0.781
hsa-miR-374a-5p	0.886	1.097	0.074	0.792
hsa-miR-28-5p	0.868	1.112	0.106	0.796
hsa-miR-26b-5p	0.886	1.071	0.076	0.816
hsa-miR-301a-3p	0.879	1.059	0.198	0.817
hsa-miR-27a-3p	0.882	1.227	0.001	0.825
hsa-miR-340-5p	0.868	1.167	0.022	0.826
hsa-miR-24-3p	0.894	1.191	0.002	0.829
hsa-miR-26a-5p	0.888	1.094	0.023	0.832
hsa-miR-29a-3p	0.898	1.141	0.033	0.833
hsa-miR-152-3p	0.878	1.113	0.073	0.850
hsa-miR-195-5p	0.865	1.084	0.035	0.858
hsa-miR-130a-3p	0.879	1.064	0.072	0.916
hsa-miR-126-5p	0.866	1.083	0.156	0.938
hsa-miR-34a-3p	0.863	1.089	0.056	0.958
hsa-miR-199a-3p	0.880	1.085	0.005	0.967
hsa-miR-22-5p	0.860	1.062	0.072	1.011

[a] MiRBase version 21 (www.mirbase.org, accessed July 25 2014).

[b] Spearman's rank correlation coefficient.

[c] Correlation to OS duration tested using Cox proportional hazards model.

[d] Stability score calculated according to the method described in Vandesompele et al. [1]

Abbreviations: OS, overall survival; C_q, quantification cycle; HR/C_q, hazard ratio per 40-C_q increase.

Supplementary Table S10. Studies identifying miRNA reference genes using unbiased approaches

Author	Year	Sample type	Storage	Samples	MiRNA reference genes
Liang [4]	2007	Normal tissue from 40 different locations	Frozen	40	**miR-103**, **miR-106b**, **miR-140**, miR-152N, miR-15b, miR-16, **miR-29a**, miR-29aN, miR-29bN, miR-30e, miR-324-3p, miR-423, mir-92, miR-92N, miR-93
Peltier [5]	2008	Normal tissue from 13 organs	Frozen	13	miR-191, miR-93, miR-106a, miR-25, miR-17-5p, miR-16, let-7a, **miR-103**, **miR-24**, miR-99a
		Normal Tumor-adjacent tissue from lung	Frozen	5	miR-191, let-7a, miR-17-5p, miR-25, miR-24, miR-320, miR-23a, miR-106a, miR-93, **miR-103**, miR-16, miR-30d
		Lung cancer	Frozen	5	**miR-103**, miR-25, miR-191, miR-24, let-7a, miR-17-5p, miR-23a, miR-106a, miR-16, miR-320, miR-93, miR-30d
Mestdagh [6]	2009	Neuroblastoma	Frozen	61	miR-125a, miR-191, **miR-425**
		Bone marrow	Frozen	11	**miR-30c**, **miR-140**, miR-328
		Normal tissue from 8 organs	Frozen	8	**let-7f**, **miR-339**, miR-572, miR-632
		T-cell acute lymphoblastic leukemia	Frozen	49	**miR-331**, **miR-361**, miR-423
		EVI-1 leukemia	Frozen	18	miR-16, **miR-140**, miR-191
Chang [7]	2010	Colorectal cancer and normal	Frozen	20	let-7a, miR-16, **miR-26a**, miR-345, **miR-425**, miR-454
Wotschofsky [8]	2011	Kidney cancer and normal kidney	Frozen	59	**miR-28**, **miR-103**, miR-106a, miR-151
Viprey [9]	2012	Peripheral whole blood and bone marrow	Frozen	98	**miR-24**, **miR-26a**, **miR-28-5p**
Current study	2014	Colorectal cancer	FFPE	197	**miR-103a-3p**, miR-152-3p, miR-132-3p, miR-27a-3p, **miR-140-5p**, miR-30b-5p, **miR-339-5p**, **miR-331-3p**, miR-374a-5p, miR-652-3p, miR-335-5p, miR-185-5p, **miR-30c-5p**, miR-151a-5p, **miR-106b-5p,** miR-199a-3p, **miR-28-5p**, **miR-425-5p**, **miR-26a-5p**, **miR-24-3p**
		Pancreatic cancer	FFPE	188	**miR-103a-3p**, miR-374b-5p, **miR-361-5p**, miR-374a-5p, let-7g-5p, **miR-28-5p**, **miR-29a-3p**, miR-301a-3p, miR-340-5p, miR-27a-3p, **miR-106b-5p**, miR-152-3p, miR-26b-5p, miR-660-5p, **miR-26a-5p**, **miR-24-3p**, miR-130a-3p, miR-23b-3p, **let-7f-5p**, miR-199a-3p,

MiRNAs in bold in the lists from the previous studies are miRNAs that were also found in the current study and miRNAs in bold from the current study are miRNAs that have been identified previously. MiRNA reference gene lists are ordered by decreasing stability in studies where stability data were available.

Abbreviations: FFPE, formalin-fixed paraffin-embedded tissue.

SUPPLEMENTARY FIGURE LEGENDS

Supplementary Figure S1. Overview of sample cohorts and associated analyses.

Supplementary Figure S2. Ratio between mean expression of the identified top four reference miRNAs and global mean miRNA expression as a function of tumor cell percentage in colorectal cancer samples.

Differences between groups were tested with Kruskal-Wallis rank-sum test.

Supplementary Figure S3. Correlation between the mean (C_q) expression of the identified top four reference miRNAs and the global mean miRNA expression in pancreas samples.

Samples were from pancreatic cancer (A), normal pancreas (B), or chronic pancreatitis (C). Correlations for paired samples were tested by means of Pearson product moment coefficient.

Supplementary Figure S4. Hierarchical clustering dendrogram of miRNA expression in methodological samples using 1-Pearson correlation and complete linkage.

Samples are named as: Project ID – Tumor number – cluster number. Project ID and tumor number are explained in Supplementary Table S1.

Supplementary Figure S5. Hierarchical clustering dendrogram of miRNA expression in methodological samples using Euclidean distance and complete linkage on raw expression values.

Samples are named as: Project ID – Tumor number – cluster number. Project ID and tumor number are explained in Supplementary Table S1.

Supplementary Figure S6. Hierarchical clustering dendrogram of miRNA expression in methodological samples using Euclidean distance and complete linkage on global mean normalized expression values.

Samples are named as: Project ID – Tumor number – cluster number. Project ID and tumor number are explained in Supplementary Table S1.

Supplementary Figure S7. Hierarchical clustering dendrogram of miRNA expression in methodological samples using Euclidean distance and complete linkage on expression values normalized by the mean of the optimal number of reference miRNAs (N=4).

Samples are named as: Project ID – Tumor number – cluster number. Project ID and tumor number are explained in Supplementary Table S1.

Supplementary Figure S8. Hierarchical clustering dendrogram of miRNA expression in methodological samples using Euclidean distance and complete linkage on expression values normalized by the mean of the two best reference miRNAs (miR-103a-3p and miR-152-3p).

Samples are named as: Project ID – Tumor number – cluster number. Project ID and tumor number are explained in Supplementary Table S1.

Supplementary Figure S9. Sample-wise comparison of high-quality mean miRNA expression in C_q (measurements with $C_q > 32$ and miRNAs with > 5% undetermined removed) and low-quality mean miRNA expression (no measurements removed).

Supplementary Figure S1

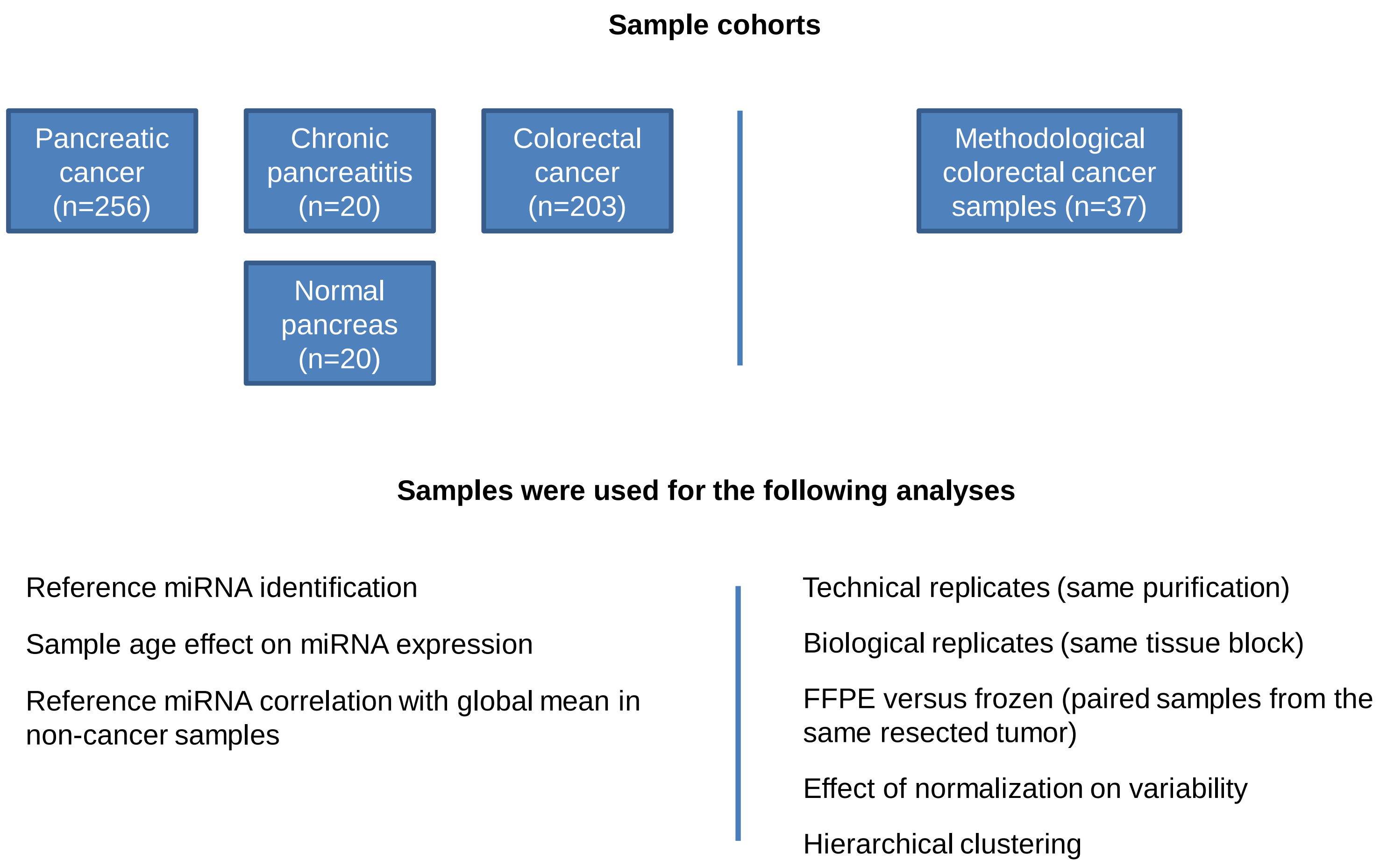

Supplementary Figure S2

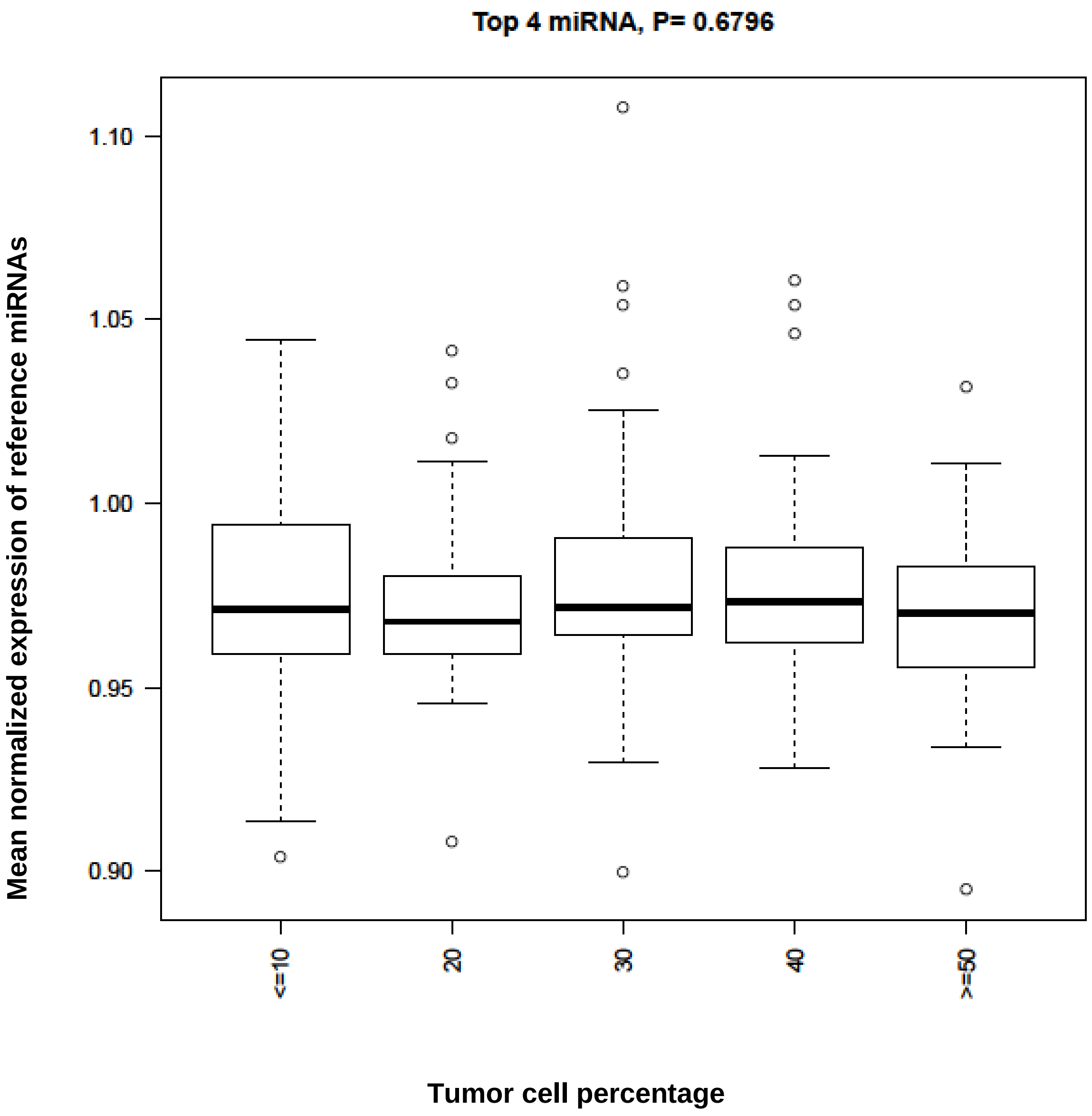

Supplementary Figure S3

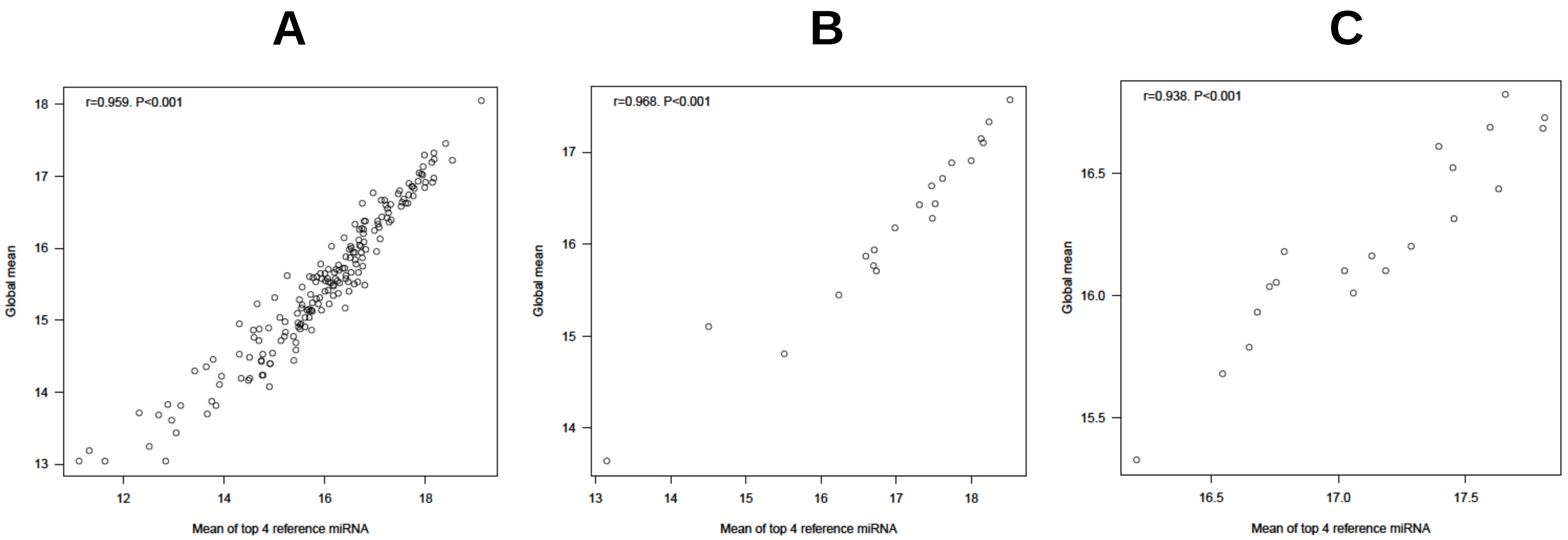

Supplementary Figure S4

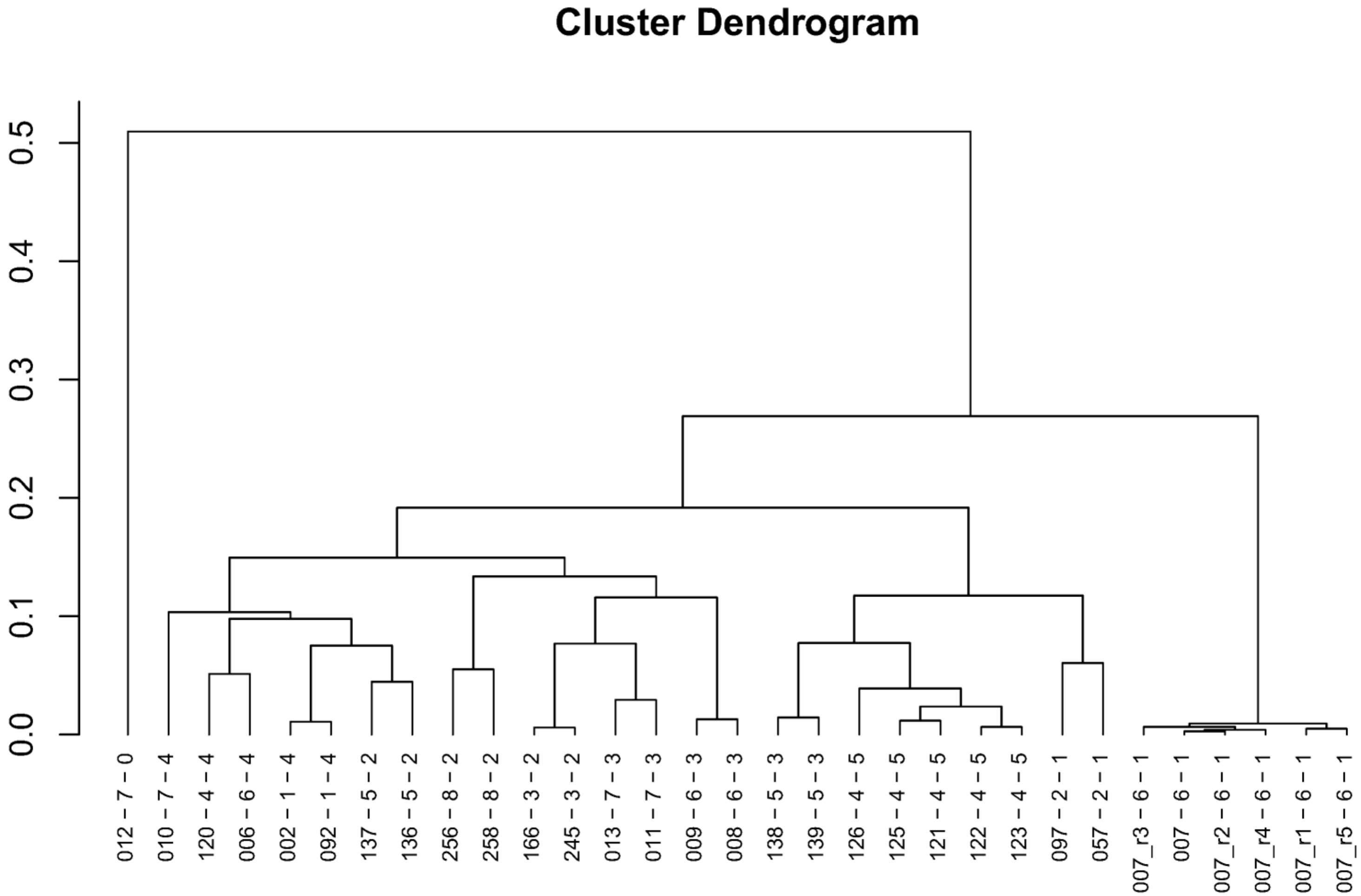

Supplementary Figure S5

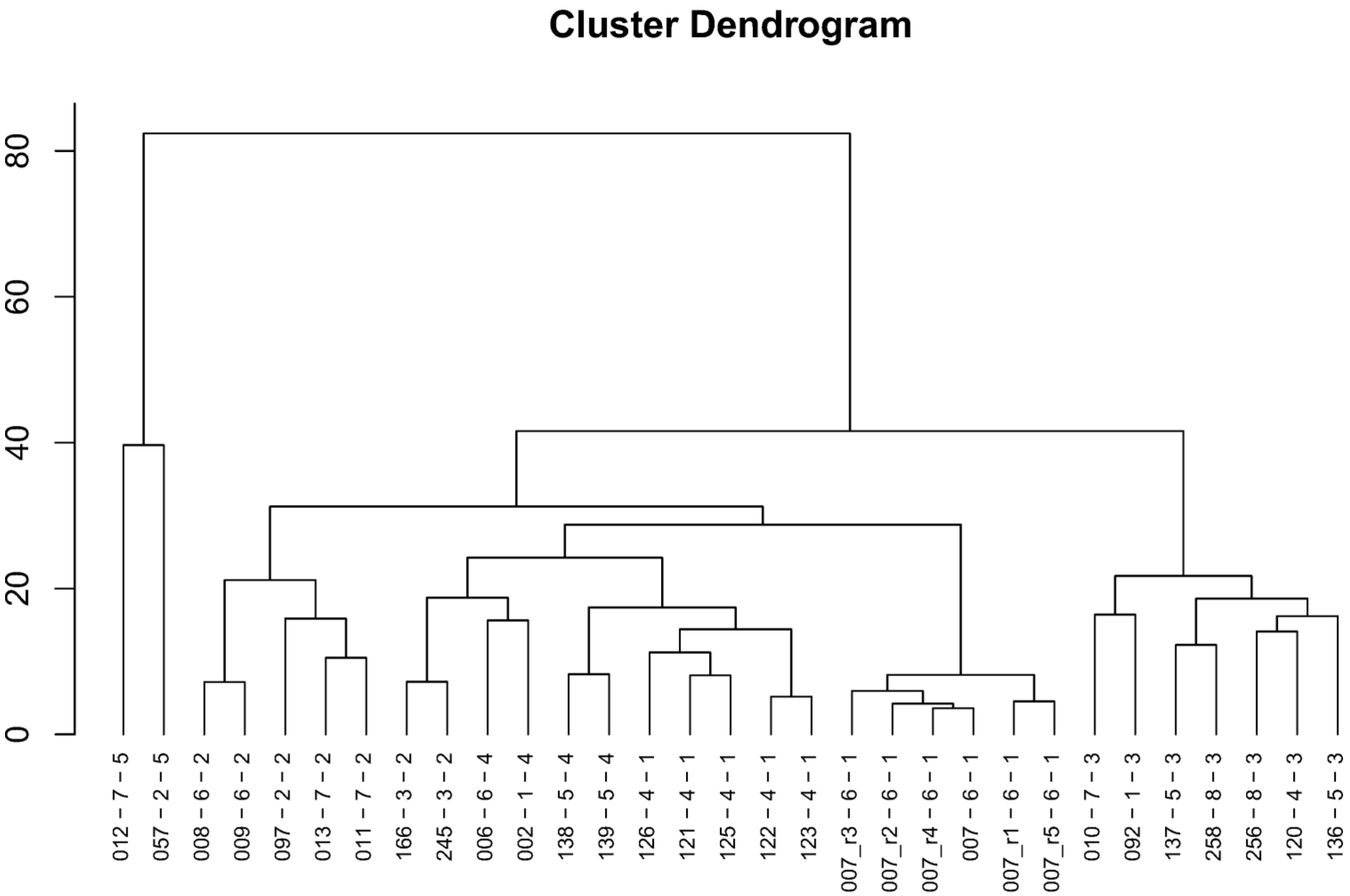

Supplementary Figure S6

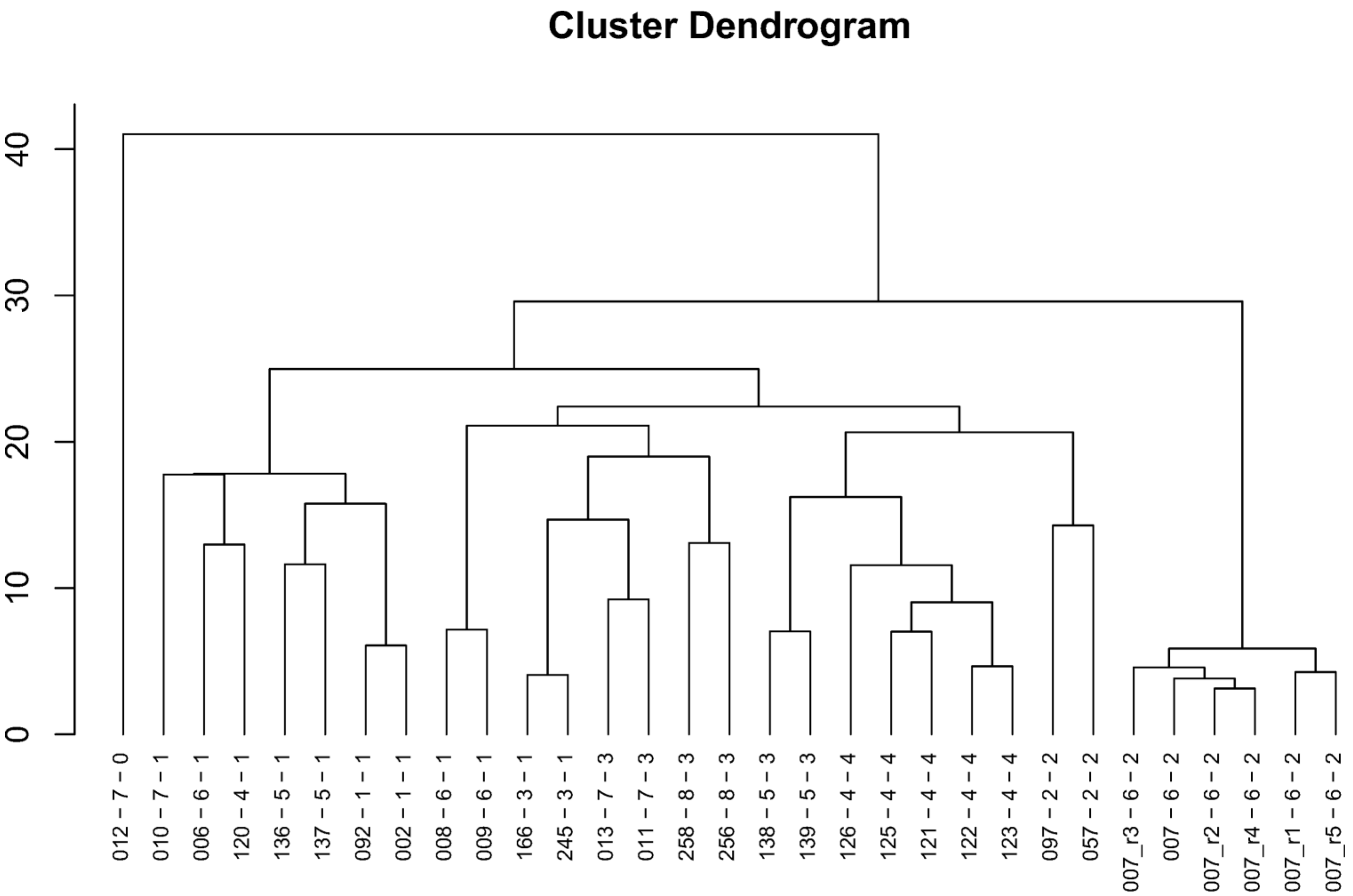

Supplementary Figure S7

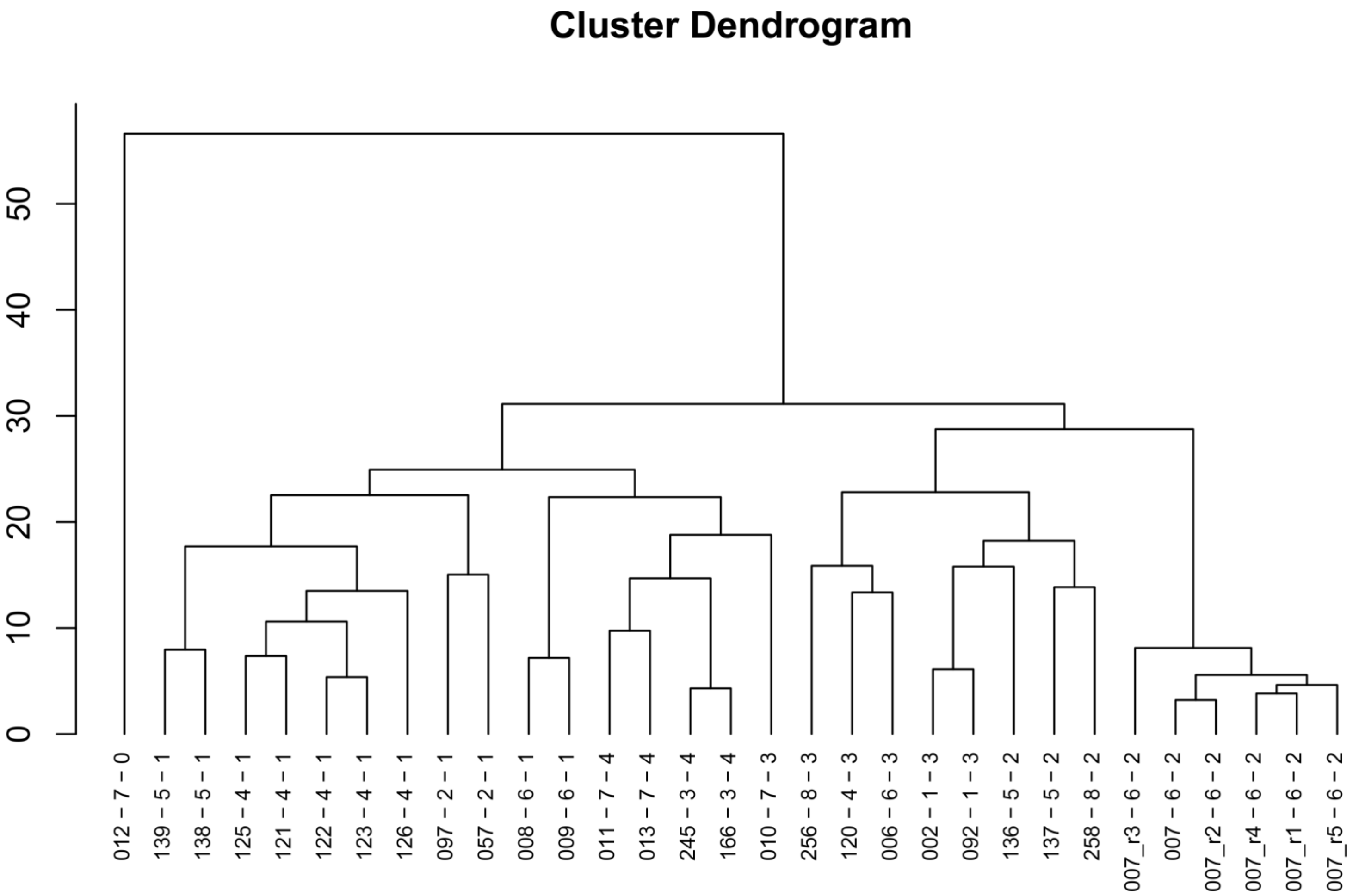

Supplementary Figure S8

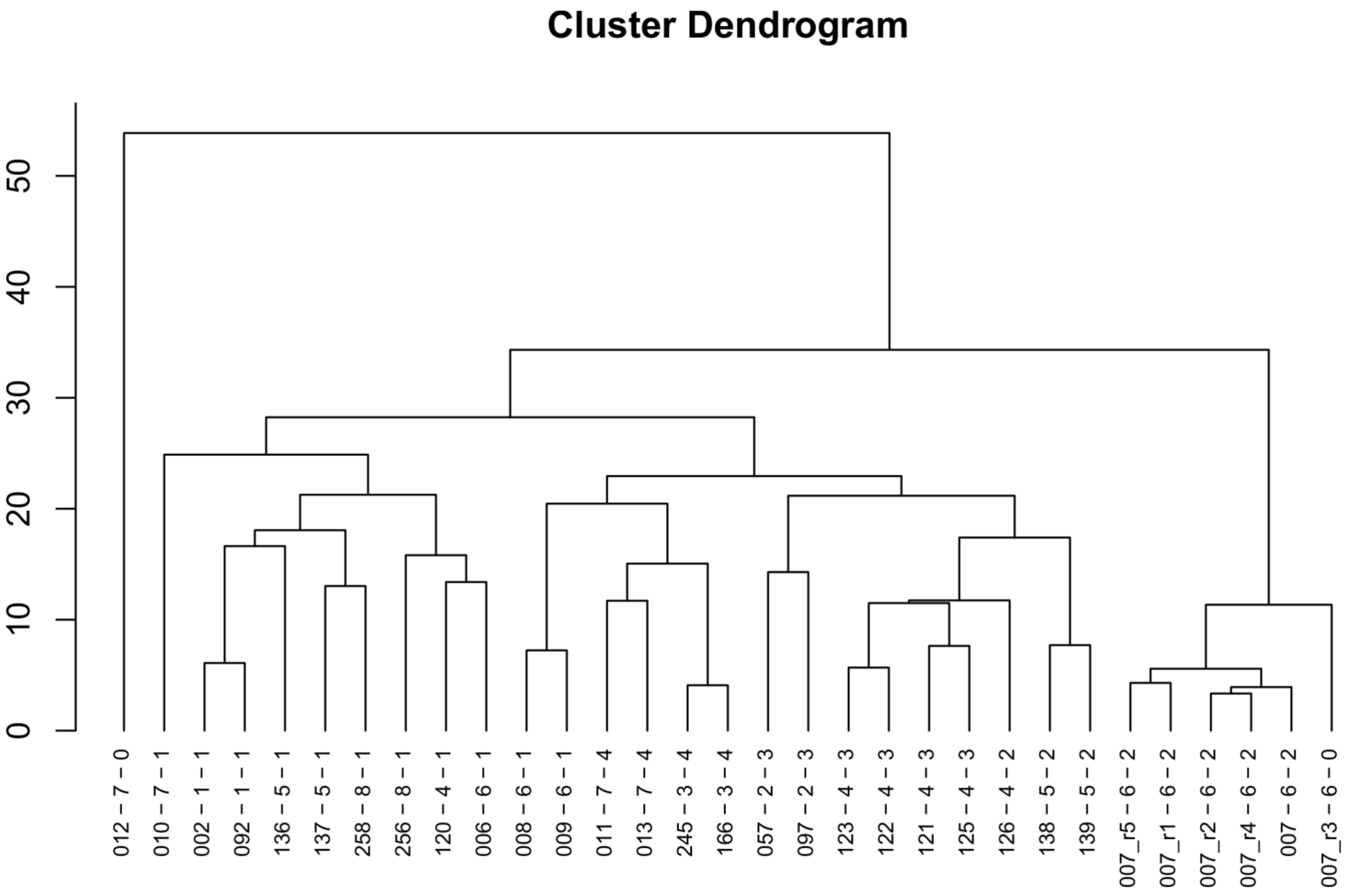

Supplementary Figure S9

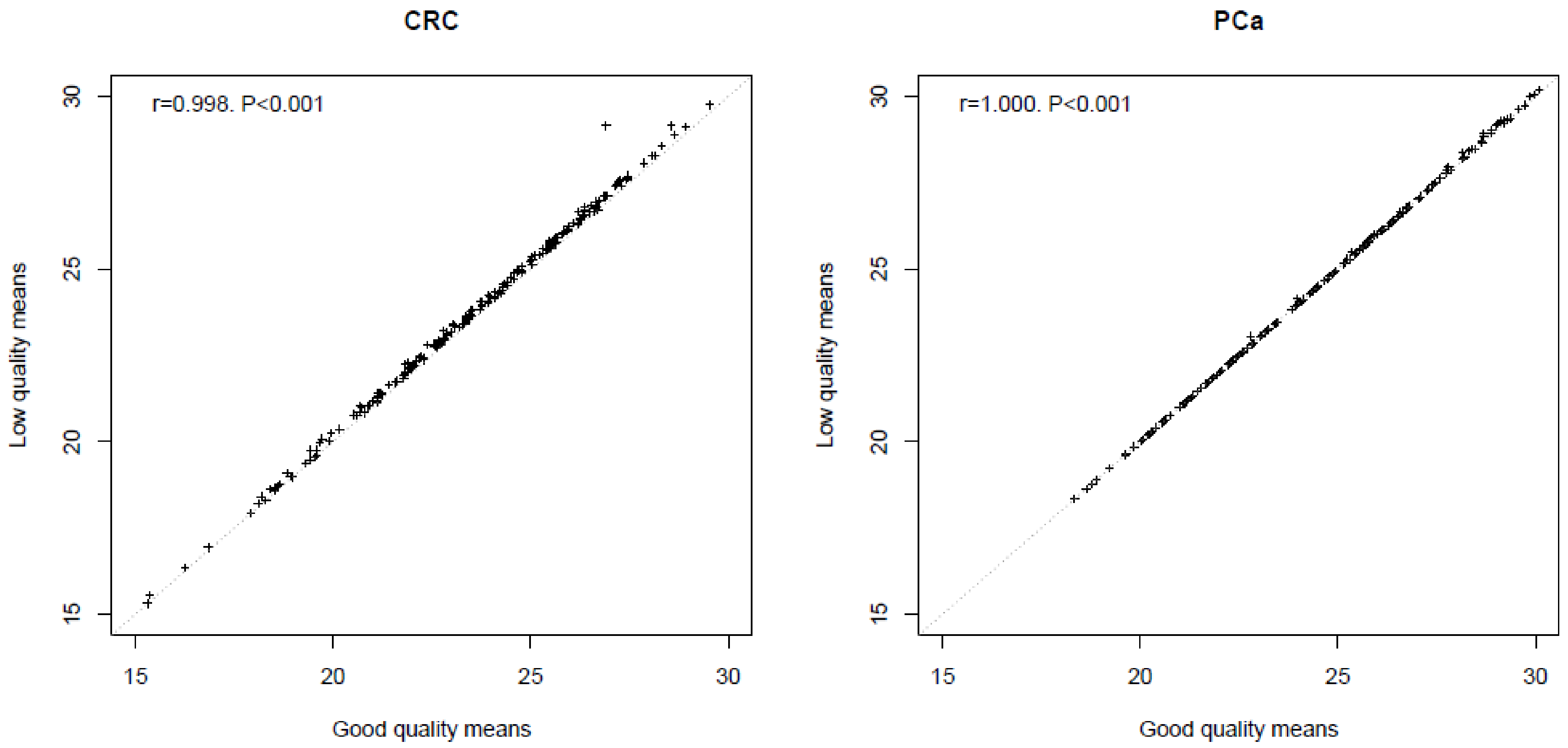

REFERENCES

1. Vandesompele J, De Preter K, Pattyn F, Poppe B, Van Roy N, De Paepe A et al. Accurate normalization of real-time quantitative RT-PCR data by geometric averaging of multiple internal control genes. Genome Biol. 2002;3(7):RESEARCH0034.

2. Boisen MK, Dehlendorff C, Linnemann D, Nielsen BS, Larsen JS, Østerlind K et al. Tissue MicroRNAs as Predictors of Outcome in Patients with Metastatic Colorectal Cancer Treated with First Line Capecitabine and Oxaliplatin with or without Bevacizumab. PLoS One. 2014;9(10):e109430.

3. Schultz NA, Andersen KK, Roslind A, Willenbrock H, Wojdemann M, Johansen JS. Prognostic microRNAs in cancer tissue from patients operated for pancreatic cancer--five microRNAs in a prognostic index. World J Surg. 2012;36(11):2699-707.

4. Liang Y, Ridzon D, Wong L, Chen C. Characterization of microRNA expression profiles in normal human tissues. BMC Genomics. 2007;8:166.

5. Peltier HJ, Latham GJ. Normalization of microRNA expression levels in quantitative RT-PCR assays: identification of suitable reference RNA targets in normal and cancerous human solid tissues. RNA. 2008;14(5):844-52.

6. Mestdagh P, Van Vlierberghe P, De Weer A, Muth D, Westermann F, Speleman F et al. A novel and universal method for microRNA RT-qPCR data normalization. Genome Biol. 2009;10(6):R64.

7. Chang KH, Mestdagh P, Vandesompele J, Kerin MJ, Miller N. MicroRNA expression profiling to identify and validate reference genes for relative quantification in colorectal cancer. BMC Cancer. 2010;10:173.

8. Wotschofsky Z, Meyer HA, Jung M, Fendler A, Wagner I, Stephan C et al. Reference genes for the relative quantification of microRNAs in renal cell carcinomas and their metastases. Anal Biochem. 2011;417(2):233-41.

9. Viprey VF, Corrias MV, Burchill SA. Identification of reference microRNAs and suitability of archived hemopoietic samples for robust microRNA expression profiling. Anal Biochem. 2012;421(2):566-72.
